# Clinical outcome of thymic lymphoepithelioma-like carcinoma: Case report of a 14-year-old male

**DOI:** 10.3892/ol.2014.2475

**Published:** 2014-08-22

**Authors:** KUNIKO SEKIHARA, YUSUKE OKUMA, HIROSHI KAWAMOTO, YUKIO HOSOMI

**Affiliations:** 1 Department of Thoracic Oncology and Respiratory Medicine, Tokyo Metropolitan Cancer and Infectious diseases Center, Komagome Hospital, Tokyo 113-8677, Japan; 2 Division of Thoracic Oncology, National Cancer Center Hospital, Tokyo 104-0045, Japan; 3 Department of Pediatrics, National Cancer Center Hospital, Tokyo 104-0045, Japan

**Keywords:** thymic carcinoma, thymoma, lymphoepithelioma-like carcinoma, chemotherapy, Epstein-Barr virus infection

## Abstract

Thymic carcinoma is a rare type of cancer, which arises from the thymic epithelium and accounts for ~1–4% of anterior mediastinal tumors in the USA. It rarely occurs in children, and is rarer among adults. Thymic lymphoepithelioma-like carcinoma (LELC) is an uncommon subtype of thymic carcinoma in children, however, it is one of the common histological subtypes of thymic carcinoma in adults. In the present study, a 14-year-old male patient presented to the Tokyo Metropolitan Cancer and Infectious diseases Center, Komagome Hospital (Tokoyo, Japan) with chest pain due to a large anterior mediastinal mass. The patient was histologically diagnosed with thymic LELC via a needle biopsy specimen, which was obtained from the primary site and indicated the Epstein-Barr virus infection, whose markers are also associated with oncogenesis. Immunohistochemical analysis demonstrated positive staining for keratin (AE1/AE3), epithelial membrane antigen, and latent membrane protein-1 and negative staining for cluster of differentiation 5. Thus, the patient was diagnosed with metastatic thymic LELC. First-line chemotherapy comprising of a cisplatin- and adriamycin-based chemotherapy regimen achieved a partial response, however, the patient succumbed within 10 months of the initial diagnosis due to rapid disease progression and refractory to subsequent cycles of chemotherapy. Thus, the current study, as well as previously reported cases, demonstrates that pediatric patients with thymic LELC continue to have a poor prognosis.

## Introduction

Thymic carcinoma is a rare type of cancer, which arises from the thymic epithelium and accounts for ~1–4% of anterior mediastinal tumors in the USA (1). A ‘rare cancer’ is defined by the Rare Cancers Europe as one that occurs in less than six patients per hundred thousand person-years (2). Thymic carcinoma is rarer still in children, constituting <1% of all types of childhood neoplasm. Thymic carcinoma was previously defined as a type C thymoma by the World Health Organization (3); however, it was reclassified as a distinct entity in 2004, as it exhibits a higher cytological atypia and poorer clinical outcome compared with thymoma (4). Thymic lymphoepithelioma-like carcinoma (LELC) is a histological subtype of aggressive thymic carcinoma, which is frequently associated with the Epstein-Barr virus (EBV) and, although it is a relatively common subtype of thymic carcinoma in adults, only 13 cases have been reported in children (5–15). Optimal chemotherapeutic regimens have not yet been determined for the treatment of thymic carcinoma (16), thus, previous case studies were conducted based on the original regimens for advanced thymoma. Previously reported cases of chemotherapeutic regimens have consisted of cisplatin- and adriamycin-based chemotherapy with response rates of 70–90% for thymomas in the prospective studies (17), compared with response rates of 20–50% for thymic carcinomas in the retrospective studies (18), which included almost all histological subtypes of squamous cell carcinoma.

In the current report, we present a case of metastatic thymic LELC in a young male (age, 14 years) who was administered with palliative-intent chemotherapy. Written informed consent was obtained from the family of the patient.

## Case report

A 14-year-old male patient presented the Tokyo Metropolitan Cancer and Infectious diseases Center, Komagome Hospital (Tokoyo, Japan) on March 30, 2010, with complaints of progressive chest and left leg pain over a two-month period. The observations from the physical examination were not significant. However, the laboratory findings revealed that the patient was anemic (hemoglobin, 12.1 mg/dl), with elevated serum levels of lactate dehydrogenase (426 IU/l), immunoglobulin (Ig) G (2,334 mg/dl), soluble-interleukin-2 receptor (1,130 IU/ml) and C-reactive protein (1.9 mg/dl). Findings from the bone marrow aspiration were normal. The results (antibody titer values) from serology were positive for EBV capsid (CA)-IgG (×2,560), EBVCA-IgA (×40), EBVCA-IgM (×10) and EB nuclear-antigen ELISA (×4.6). A computed tomography (CT) scan of the chest revealed an 8.5×5.6-cm mass in the left anterior mediastinum, and magnetic resonance imaging (MRI) demonstrated multiple vertebral metastases and a metastatic tumor of the sacral bone, which was compressing the intrapelvic organs. Histopathologic examination of a needle biopsy specimen from the anterior mediastinal mass revealed a neoplasm, which was composed of anastomosing cords or nests of carcinoma cells interspersed with lymphocytes. The tumor cells exhibited large vesicular nuclei with distinct nucleoli. Immunohistochemical (IHC) staining was also positive for keratin (AE1/AE3), epithelial membrane antigen and latent membrane protein (LMP-1), however, was negative for cluster of differentiation 5. Based on these findings, the patient was diagnosed with LELC, stage IVb according to the Masaoka-Koga staging system (19).

The patient was treated with a chemotherapy regimen as follows: Doxorubicin (30 mg/m^2^, day 1), vincristine (1.5 mg/m^2^, day 1), cyclophosphamide (1,200 mg/m^2^, day 1) and cisplatin (90 mg/m^2^, day 2) every for three cycles, each lasting three weeks. A subsequent CT scan demonstrated that the tumor had reduced by 54% according to the Response Evaluation Criteria In Solid Tumors guidelines (version 1.1) (20). However, no further regression of the tumor was achieved following two additional cycles and the patient developed a neoplastic fever. The patient was switched to the second-line chemotherapy, consisting of a daily dose of 100 mg oral S-1, administered two-weeks-on and one-week-off. The patient’s fever reduced during the first cycle, however, multiple hepatic metastases were observed via CT scan following a further cycle and the patient developed a neoplastic fever.

For the third-line chemotherapy, the patient was treated with three, weekly cycles of carboplatin (CBDCA; area under the curve=6, day 1) and paclitaxel (PTX; 200 mg/m^2^, day 1). Initially, the hepatic metastases regressed and the fever subsided. However, following two cycles of the CBDCA/PTX regimen, the multiple hepatic metastases became exacerbated and the patient’s temperature increased. No further chemotherapy was prescribed due to prolonged thrombocytopenia and the patient received best supportive care. The patient succumbed to the disease 10 months after the initial diagnosis.

## Discussion

Thymic carcinomas were initially reported in 1977 by Shimosato *et al* (21). The WHO previously classified all neoplasms arising from the thymic epithelium as thymomas, however, these neoplasms were reclassified in 2004 as separate from thymomas (4). Thymic carcinomas exhibit more aggressive biological behavior and lower clinical success rates when compared with thymomas, and they are markedly less frequently associated with paraneoplastic syndrome. In the 2004 WHO classification, 13 subtypes of thymic carcinoma were recognized, including squamous cell, lymphoepithelioma-like, mucoepidermoid and neuroendocrine carcinoma (4). Squamous cell carcinoma and LELC are relatively common subtypes of adult thymic carcinoma. However, to the best of our knowledge, only 13 cases of LELC have been reported in children (Table I) (5–15). EBV is hypothesized to be associated with LELC and LMP-1 is considered to be a predominant EBV oncoprotein (7,24–26). In the current case, LMP-1 was demonstrated in the tumor cells using IHC staining.

The optimal treatment is considered to be complete surgical excision. However, thymic LELC often invades adjacent structures and metastasizes to distant organs (such as, the lungs, liver, bone and lymph nodes) at the point of initial diagnosis, indicating that it exhibits high-grade atypia, which results in a poor prognosis. Thymic carcinoma appear to be clinically indolent and aggressive with regard to clinical behaviour and chemotherapy sensitivity. In the current case, the patient’s LELC appeared to be clinically aggressive, however, the first-line chemotherapy achieved a partial response. Due to the rarity of the disease, no standard and definitive systemic chemotherapy has been established for metastatic thymic carcinoma. According to an empirical investigation, the conventional chemotherapeutic regimens for thymoma, which are cisplatin- and anthracycline-based triplet or quartet regimens, are considered to be ADOC (cisplatin, doxorubicin, vincristine and cyclophosphamide) or PAC (cisplatin, cyclophosphamide and adriamycin) (16). The response rate to ADOC for thymic carcinoma was found to be 50% in a previous retrospective study, however, the National Comprehensive Cancer Network clinical guideline (27) recommends the CBDCA/PTX regimen, which was appraised by a phase II study and achieved a response rate of 21.7%. In the present study, the patient was treated with a modified ADOC-based regimen for pediatric stage IV neuroblastoma, termed as a novel A1 regimen [vincristine (day 1, 1.5 mg/m^2^), cyclophosphamide (day 1, 1,200 mg/m^2^), thermal enhancement of pirarubicin-adriamycin (day 3, 40 mg/m^2^) and cisplatin (day 5, 90 mg/m^2^) every four weeks] and subsequently changed to cisplatin dose-dense chemotherapy, which achieved a partial response (28). Oral S-1 has previously been reported to be useful as a second-line (or later) chemotherapy (22). Furthermore, CBDCA/PTX was found to be efficacious for thymic carcinoma as a first-line (or later) chemotherapy in a retrospective study (22). In the present case, oral S-1 and CBDCA/PTX were considered to be beneficial for palliation.

In conclusion, chemotherapy with palliative intent is an option for temporarily relieving symptoms by reducing the tumor size; however, thymic LELC exhibited highly malignant atypia and, therefore, the patient in the current study had a poor prognosis and succumbed to the disease 10 months following initial diagnosis.

## Figures and Tables

**Table I:** Reported cases of pediatric thymic lymphepithelioma-like carcinoma.

First author, year (Ref.)	Age (years)/Gender	Stage^a^	Metastasis	Treatment	Outcome
Wick, 1982 (4)	4/Male	N/A	None	None	Died within 2 months
Marino, 1985 (5)	14/Male	N/A	Intrathoracic	N/A	N/A
Fuji, 1993 (6)	13/Female	IVb	SPC node	Surg + CT + RT	Died within 22 months
Ilhan, 1994 (7)	13/Female	IVb	SPC node	Surg + CT + RT	Alive after 3 years
Niehues, 1996 (8)	14/Male	III	Invasion of the lung and pericardium	Surg + CT + RT	Alive after 12 years
Stéphan, 2000 (9)	12/Female	IVb	Lung, bone	CT + RT	Died within 16 months
Di Cataldo, 2000 (10)	11/Male	IVb	SPC node	Surg + CT + RT	Died within 1 year
Yaris, 2006 (11)	16/Female	IVb	Lung	CT + RT	Died within 15 months
Hsueh, 2006 (12)	14/Male	II	Extension to right pleura	CT + RT	Died within 10 months
	10/Male	III	Invasion of lung	Surg + CT + RT	Died within 11 months
Tacyildiz, 2007 (13)	10/Male	III	Invasion of vessels	CT + Surg + RT	Alive after 1 year
Killis-Pstrusinska, 2008 (14)	16/Male	IVa	Pleural dissemination	CT	Died within 11 months
Present case	14/Male	IVb	Bone	CT	Died within 10 months

a Masaoka-Koga staging system.

CT, chemotherapy; RT, radiotherapy; Surg, surgery; SPC, supraclavicular; N/A, not available.
